# Evaluating erroneous offside calls in soccer

**DOI:** 10.1371/journal.pone.0174358

**Published:** 2017-03-23

**Authors:** Stefanie Hüttermann, Benjamin Noël, Daniel Memmert

**Affiliations:** Institute of Training Science and Sport Informatics, German Sport University Cologne, Cologne, Germany; Universidade de Tras-os-Montes e Alto Douro, PORTUGAL

## Abstract

The ability to simultaneously attend to multiple objects declines with increases in the visual angle separating distant objects. We explored whether these laboratory-measured limits on visual attentional spread generalize to a real life context: offside calls by soccer assistant referees. We coded all offside calls from a full year of first division German soccer matches. By determining the x-y coordinates of the relevant players and assistant referee on the soccer field we were able to calculate how far assistant referees had to spread their visual attention to perform well. Counterintuitively, assistant referees made fewer errors when they were farther away from the action due to an advantageous (smaller) visual angle on the game action. The pattern held even when we accounted for individual differences in a laboratory-based attentional spread measure of ten of the assistant referees. Our finding that errors are linked to smaller visual angles may explain the complaints of fans in some situations: Those seated directly behind the assistant referee, further from the players, might actually have it easier to make the right call because the relevant players would form a smaller visual angle.

## Introduction

Referees often take the blame for mistaken calls, but in some cases they may be at an unfair disadvantage. The perception of spatially disparate but simultaneous events might exceed the limits of their ability to spread attention; in laboratory tasks, the ability to make accurate judgments about spatially disparate objects diminishes as the visual angle between those objects increases [1].

Offside calls in soccer provide an ideal test for the importance of spatial attention in a real world context. According to FIFA rules, a player is offside when he/she is in the opposite team’s half of the pitch and closer to the opponents’ goal line than both the ball and all but the last defender (typically the goalkeeper) in the exact moment the ball is passed forward by a teammate. Assistant referees make errors on approximately 20–26% of offside calls [2–4], and many factors contribute to such errors (see [5]). Among others, assistant referees make more errors when they are running to maintain the appropriate position than when they are walking or stationary [4]. In addition, a distorted view arises for the assistant referee on the location and the position of the players (receiver and defender) to one another, as soon as the position of the assistant referee drifts from the offside line (cf. flash-lag effect and optical-error-hypothesis; [6,7]).

When determining whether an offensive player is offside, an assistant referee must attentively select the relevant players from other players, and has to simultaneously focus attention on objects and their spatial/temporal relations at spatially disparate regions of the field. In the case of an offside decision the relevant players are the passer, receiver, and second last defender during the moment of the pass. That is, an assistant referee has to simultaneously identify the moment a ball is passed forward (attention to the passer) and the relative position of the receiver and second last defender (attention to the area/players close to the offside line). Consequently, limits on the ability to focus attention selectively at two locations should influence performance.

Yet, only one previous study has explored the role of visual angle and consequently the role of the players’ separation in error rates [8]. In the one study, error rates did not differ as a function of visual angle (discretized into angle categories of 0–15°, 16–30°, 31–45°, 46–60°, 61–75°, 76–90°). In that study, the vast majority of the errors analyzed (88%) were *misses*, cases in which the assistant referee should have made an offside call but did not. However, such non-calls do not provide an optimal test of the role of visual angle for several reasons. Most importantly, assistant referees tend to have a conservative bias when making calls; when in doubt, they tend not to make a call and always decide in favor of the offensive player [2]. Consequently, when an assistant referee fails to make an offside call and the offensive player actually was offside, we cannot determine whether they failed to perceive the situation as offside or were uncertain enough that they chose not to make an overt error. For that reason, we chose to focus exclusively on overt offside calls, those in which the assistant referee raised his flag. Given the conservative bias in making calls, we can be fairly certain that the majority of flagged calls were due to failures of visual attention rather than to a general tendency to make a call when uncertain. That is, the false positives constitute cases in which the assistant referee misperceived the situation and by analyzing flagged calls we can more readily determine whether there is a link between the visual angle separating the critical players and the probability of an error. However, to scrutinize if the limited visual angle is indeed a decisive factor for wrong decision-making in offside situations, we considered the two spatial distances that determine the angle: the spatial separation of the assistant referee between the passer and defender on the offside line (horizontal attentional spread) as well as the spatial separation in depth between the assistant referee and both of those two players (attentional spread in the depth). We expected assistant referees to show greater error rates in their decision-making with increasing visual angle. That is, we expected a larger number of errors with a larger separation between players (i.e., larger horizontal attentional spread). Furthermore, we assumed to find higher error rates for smaller separations between the assistant referee and the players in depth (i.e., smaller attentional spread in depth) due to concomitant greater visual angles.

## Study 1

We examined all offside calls from a complete season (2010/2011) of first German division soccer games (306 games). To be included in our analysis, the two involved offensive players, the defensive player as well as the assistant referee must have been clearly visible in the video. The initial coding yielded 355 flagged offside calls from 211 games. Assistant referees are supposed to position themselves at the offside line so that they can better judge the left/right position of the relevant offensive and defensive players. If the player deviates substantially from that position, their calls might be influenced not just by left/right judgments but also by differences in the relative depth of the players (see [3,6,7]). Oudejans et al. [7] observed perceptual illusions in which assistant referees were positioned more than 1m from the imaginary offside line. However, considering that there was no case in our data set in which the distance between the assistant referee and the offside line was bigger than 1m we did not have to control for that confound. The mean deviation from the offside line was 0.79m.

An offside call depends on the relative positions of three players and the ball: the offensive player who passes the ball (passer), the offensive player who receives the ball (receiver), and the defensive player who is second closest to the goal line (defender; typically, the goalkeeper is the closest to the goal line). If the receiver is further from the goal or as far away as the defender at the instant the passer kicks the ball forward, the play is onside. If the receiver is closer to the goal than the defender when the passer kicks the ball, the play is offside. In order to make the correct call, the assistant referee must determine the relative positions of the receiver and last defender at that moment. A correct call depends on focusing attention on both the passer and the receiver/defender. Consequently, the crucial visual angle always involves the passer and one of the other two players. Considering that assistant referees are always required to position themselves at the height of the last defense player the maximum visual angle to their left side is between the passer and the defender regardless of whether it is an onside or an offside situation. In addition to determining the assistant referee’s visual angle between passer and defender we have determined the y separation in depth between the assistant referee and the passer (or more specifically the y separation between the passer and the sideline) as well as between the assistant referee and the defender.

Each offside call was analyzed by using utilius® easyinspect. This program converts two-dimensional frames from soccer videos into three-dimensional models, allowing extraction of the relevant player positions. The program performs the conversion by relying on the known distances among the boundaries of the playing field. For our analyses, we paused the video and analyzed the critical offside situation at the moment the ball left the passer’s foot. We coded the x-y coordinates of the second last defender, passer, receiver, and assistant referee using known landmarks on the field, excluding those offside calls for which we lacked the necessary fixed references (n = 48). In our coding scheme, the x dimension ran between the goals and the y dimension ran from sideline to sideline. We then set the assistant referee’s position to 0,0 and adjusted the relative coordinates for the players. From these coordinates, we calculated the assistant referee’s visual angle by calculating the separation between the passer and defender (horizontal attentional spread) as well as the separation in depth between the assistant referee (or rather the sideline) and both players (attentional spread in depth).

## Results and discussion

Even though our database included all calls from an entire season of a top league, the set of calls in our database cannot be treated as independent. Each assistant referee worked in multiple games, but the data set does not include enough observations per game and per assistant referee to examine the extent of non-independence of the calls. Given that assistant referees may differ in their bias to make calls and that those biases might vary across games (e.g., based on the aggressiveness of the play), treating each call as independent of other calls might not be justified. This issue has largely been ignored in other analyses of decision-making errors in sports, with papers regularly treating each observation as if it was independent of all other observations. Given this potential non-independence, the assumptions underlying null-hypothesis significance testing are possibly violated. If we had sufficient data to construct a full multi-level model incorporating games and assistant referees, we would be better able to account for any possible non-independence. Unfortunately, each game and assistant referee has too few calls to make such an analysis possible. Consequently, we present all of our data graphically and provide complete descriptive statistics rather than conducting null-hypothesis significance tests. All of our analyses should be treated as exploratory.

**Fig 1:**
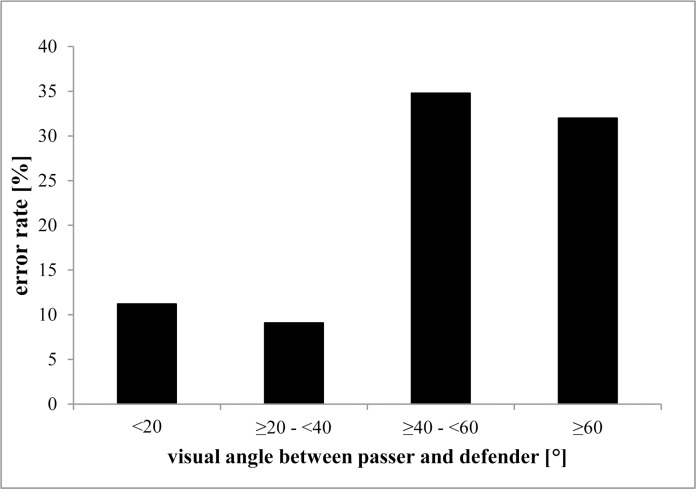
Averaged error rate as a function of degrees of assistant referees’ visual angle between the passer and the defender. Note that the number of observations contributing to each bar varies.

Out of the 355 coded offside calls, assistant referees made a total of 49 errors. This error rate (14%) is in line with estimates that up to 20% of offside calls are incorrect [3]. Fig 1 constitutes the offside calls as a function of the assistant referee’s visual angle between the passer and defender. Consistent with the prediction that error rates should increase as the visual angle increases, errors were more frequent with large visual angles, and the average visual angle for mistaken calls (*M* = 30.0, 95% CI 22.6, 37.4) was substantially greater than for correct ones (*M* = 18.6, 95% CI 16.6, 20.6). Only 11% of calls (33/302) were erroneous when the visual angle was less than 40°, but 30% (16/53) were erroneous for visual angles over 40°. These results are consistent with laboratory evidence for diminished performance with increasing visual angle and with measured limits of visual attentional spread capacities above 40° of visual angle [1]. They are inconsistent, however, with the lack of a relationship between visual angle and error rates in the only other study to examine offside calls as a function of visual angle [8]. Note, though, that 88% of the errors in the earlier study were missed calls (assistant referees did not make a call when they should have) whereas all of our analyzed errors were incorrect calls. Given that it is difficult to differentiate between cases in which an assistant referee failed to perceive the situation as offside and cases in which an assistant referee was not certain enough to raise his flag, we cannot rule out that the vast majority of non-flag errors were not related to failures of perception/attention. Consequently, flagged calls seemingly better allow to test questions on limited attentional spread performance that motivated our study. One approach is to be in direct verbal contact with the assistant referees and to create situations, in which they could be asked to think loud and provide running commentary of the perceived in-game information and the processes, on which basis they made a decision (e.g., [9]). The idea behind this is to analyze and notice non-flag situations in future research, if assistant referees did not raise their flag in such situations, because they consciously saw that it was not an offside situation or they did not raise the flag due to a level of uncertainty.

When considering the spatial separation between the passer and defender (required horizontal attentional spread), it becomes clear that the average separation for incorrect and correct calls was roughly comparable (correct calls: *M* = 6.97m, 95% CI 6.19, 7.74; incorrect calls: *M* = 7.84m, 95% CI 5.45, 10.22). This means that the relationship between the visual angle and error rates cannot be explained solely via the separation between the players, but possibly also via the separation between the assistant referee and the players in the depth.

**Fig 2:**
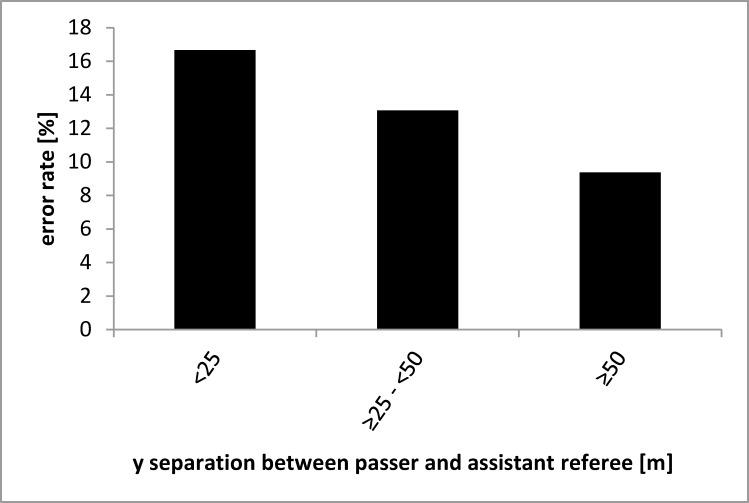
Averaged error rate as a function of the separation in depth between the assistant referee and the passer in meters. Note that the number of observations contributing to each bar varies.

**Fig 3:**
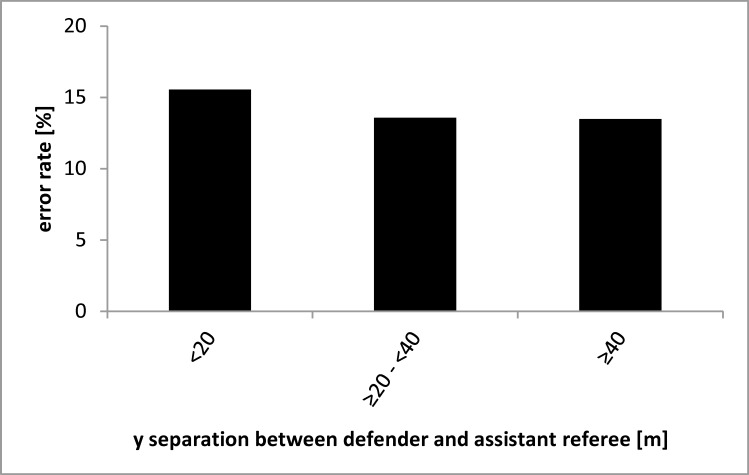
Averaged error rate as a function of the separation in depth between the assistant referee and the defender in meters. Note that the number of observations contributing to each bar varies.

Hence, we next examined whether the y separations between the assistant referee (or more specifically the sideline) and the passer as well as between the assistant referee and the defender affect the assistant referee’s performance (attentional spread in depth). As it is shown in Figs 2 and 3, error rates increased with decreasing y separations between the assistant referee and players. This indicates that the attentional spread in the depth does not negatively affect the assistant referee’s decision making; rather, a greater separation between the assistant referee and the players in the depth seems to be of advantage because of the associated decreased visual angle.

## Study 2

Although each game has two assistant referees, only one of them can make a call on a given play—each assistant referee is responsible for offside calls on one end of the pitch, and they maintain a position corresponding to the offside line (the defender’s position). Of the 50 assistant referees involved in the games included in our analyses in Study 1, 31 made calls in which all relevant players (passer, receiver, second last defender) and the respective assistant referee were clearly visible. To determine whether the decision performance of assistant referees on the field can be compared to generally existing visual/attentional limitations, we subsequently recruited ten of these 31 assistant referees who were willing and able to come to the lab to determine their maximum visual angle using a laboratory-based attentional spread measure [10]. This measurement task has been used in a number of research studies (for a review see [11]) and has the potential to precisely quantify participants’ attentional spread or visual angle when two stimuli/events have to be perceived simultaneously. If a relationship between false decisions of assistant referees on the pitch and a limitation of their attentional spread in the lab will be found, consideration should be given to possible training concepts in the lab to improve performances on the soccer field in the long term.

**Fig 4:**
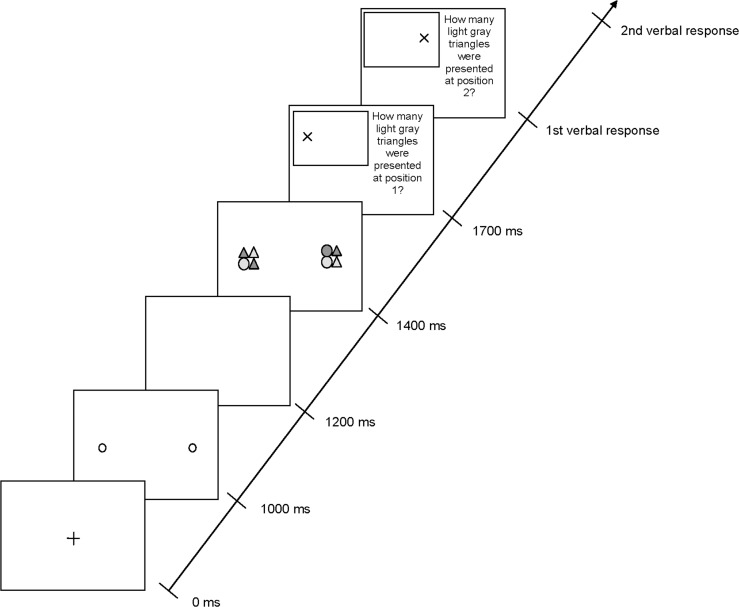
Sequence of events in a trial with stimuli along the horizontal meridian. (Note: Reprinted from [10] under CC-BY license).

The assistant referees participating in Study 2 were responsible for 157 of the 355 calls analyzed in Study 1. They averaged 11.60 (*SD* = 2.50) years of experience as First German Division assistant referees and were on the FIFA list (*M*_age_ = 38.10 years, *SD* = 1.29 years). The study was approved by the Ethics board of the German Sport University Cologne. Written informed consent was obtained from each subject prior to participation in the study in accordance with the principles of the Declaration of Helsinki 1975. Each assistant referee was tested individually in a single session lasting approximately 20 minutes. They sat approximately 50cm from a 24-inch display (resolution: 1920 x 1080 pixel, controlled by an Esprimo 710 3.3 GHz-Core i3-3220 computer) to perform the attentional spread measure task ([10], see Fig 4). The task was programmed in E-Prime 2.0 (Psychology Software Tools, Pittsburgh, PA), and participants responded using a keyboard. They were required to perceive and identify two peripheral stimuli simultaneously. After reading instructions and having any questions answered, participants completed six blocks of 48 trials (with each block followed by a 30s break), for a total of 288 trials. On each trial, a pair of stimuli appeared along one of four meridians (0°/180°, 45°/225°, 90°/270°, 135°/315; in total one horizontal, one vertical, and two diagonal meridians), positioned symmetrically around the screen center. (Please note that for analyses, data from the two diagonal meridians were combined). The meridian and separation between stimuli varied randomly across trials. As a consequence, the visual angle (calculated from the horizontal, vertical, or diagonal attentional spread and the distance between the participants and the test area) varied across trials as well and ranged from 5° to 57.5° in 7.5° steps.

Each stimulus (corresponding to a size of 8.38°) was composed of four elements (each 3.97°, with a gap of 0.44° between the elements), which could be filled circles or triangles that were light or dark gray. Each element could be any combination of form (circle, triangle) and shading (light gray, dark gray), selected at random on each trial with the constraint that each stimulus had an equal probability (20%) of including zero, one, two, three, or four light gray triangles. Assistant referees had to identify the number of light gray triangles in each stimulus, a task that requires attentive processing of a conjunction of shape and color [12].

In the laboratory-based attentional spread measure (cf. [1,10]), responses were counted as correct only if participants reported the right number of light gray triangles in both stimuli. Visual attentional spread was defined as the largest stimulus separation (measuring started from the smallest stimulus separation of 5°) at which each assistant referee reliably identified the number of light gray triangles in both stimuli on at least 75% of the trials (cf. [1]).

## Results and discussion

Averaging across meridians, the maximum visual angle was 33.25° (*SD* = 3.05°). If the separation between both stimuli exceeded this threshold, performance decreased. A repeated measures ANOVA revealed that attentional spread differed as a function of meridian, *F*(2,18) = 4.492, *p* = .026, *η*_*p*_^2^ = .333, and that it was largest along the horizontal meridian (*M* = 37.25°, *SD* = 7.12°; diagonal: *M* = 33.50°, *SD* = 5.92°; vertical: *M* = 29.00°, *SD* = 4.28°). Although we tested all meridians, for the following analyses, we focused exclusively on the horizontal meridian which is most likely related to the horizontal attentional spread measured in Study 1 (i.e., the separation between the passer and defender). The visual angle of the assistant referees was calculated via the horizontal attentional spread and their distance to the test area (i.e., attentional spread in the depth). (One needs to note here that opposed to Study 1, the attentional spread in the depth always stayed the same and the measured visual angle was only varied by the alteration of the horizontal attentional spread.)

**Fig 5:**
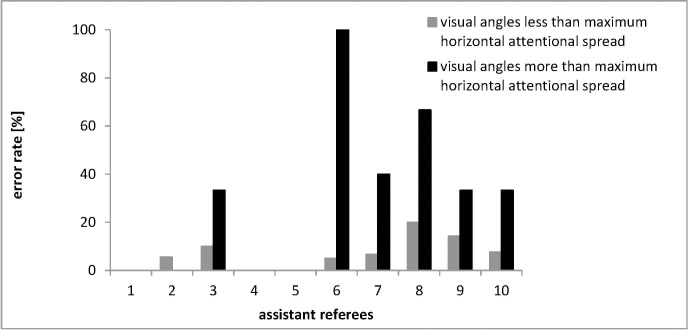
All offside calls made by the ten assistant referees who participated in the laboratory-based attentional spread task plotted as a deviation from each assistant referee’s maximum visual angle. The error rate for the assistant referees’ decision requiring visual angles less than the assistant referees’ individual maximum visual angle measured in the laboratory task is presented in gray and for situations requiring visual angles greater than the individual maximum visual angle in black. The X-axis shows each assistant referee’s number and the Y-axis shows the assistant referees’ error rate.

Although the pattern of errors across all assistant referees is clear, people differ in their ability to focus attention on separated objects. To explore whether the pattern of results presented in Study 1 holds true even when accounting for individual differences in limitations of attentional spread, in Study 2, we recoded the visual angle associated with each offside call for each of the ten assistant referees who participated in the laboratory-based attentional spread task by subtracting the estimate of their measured visual angle (centering the calls on that threshold). Of the 157 calls made by these ten assistant referees, 88.54% (139 calls) were correct and 11.46% (18 calls) were incorrect. Fig 5 plots all of the calls made by each of the ten assistant referees as a deviation from their individually measured visual angle. The gray graphs constitute cases in which the measured visual angle exceeded that assistant referee’s attentional threshold for visual angle in the laboratory task and the black graphs cases in which the measured visual angle undershot that threshold. (Note: The pattern was the same if we plotted the percentage difference from the threshold rather than the absolute difference in visual angle.) The data from these ten assistant referees are consistent with the results from all 31 assistant referees presented in Study 1—erroneous offside calls are associated with limited attentional spread dependent on the required visual angle.

## General discussion

Visual angle is associated with error rates in offside decisions by soccer assistant referees, just as it is for laboratory measures of attentional spread. Assistant referees are more error prone when the visual angle that is required in order to perceive the relevant players increases. Smaller separations in depth between the assistant referee and the players negatively affect the assistant referee’s decision-making, possibly due to an associated greater visual angle.

The analysis of 355 offside situations in Study 1 showed that error rates were lower when the relevant players were separated by visual angles smaller than about 40° compared to scenes requiring larger angles. Although we lack sufficient data for a full hierarchical model to separate the relative contributions of visual angle and other variables (e.g., separation in depth), the pattern is consistent with reduced performance as the visual attentional spread requirements increase, just as it is shown for laboratory-based measures (e.g., [1]).

In our data set, a handful of assistant referees made most of the calls and errors, with some assistant referees contributing few calls or errors. That pattern highlights the importance of considering the independence of observations in studies of judgments in sports. Many studies just combine across all observations to conduct statistical analyses, but doing so for the purposes of null-hypothesis significance testing is inappropriate when the observations are not independent. Hierarchical analyses that consider both the judgment and the person making the judgment would be appropriate, but are only possible when each participant contributes enough observations. For those reasons, we instead plotted all of the data, separating the observations for each assistant referee, thereby giving a complete description of the data set. Future studies could potentially analyze a substantially larger set of games, with many observations from each assistant referee, in order to more fully model the relationship between visual angle and decision-making in offside calls. A larger volume of decisions by individuals would expand the discussed explorative approach and would allow appropriate analyses.

In Study 1, we did not systematically vary the visual angle (or other related factors such as the separation in depth between the assistant referee and the players) to determine the effect on assistant referees’ error rates, so we cannot claim that greater visual angles cause greater error rates: It is possible that a third factor, something about how the play takes form, could contribute both to greater visual angles and to greater error rates. For example, assistant referees might adopt a different strategy when the visual angle is larger, leading to higher error rates. Moreover, given that we only coded overt calls, it is possible that shifts in bias—the willingness to make calls—might vary systematically with visual angle. Still, the pattern of errors observed in actual performance aligns well with the effects of angular separation on performance measured in the lab, suggesting that errors become more prevalent as the visual angle exceeds the limits of attentional spread (cf. [7,13]).

There is a number of aspects, which have possibly influenced the decision behavior of (assistant) referees, but which were not monitored as a part of this study. It was not considered, for example, that decisions often depend on the time in the match (e.g., usually fewer offside calls are made in the first half than in the second half, [8]). Furthermore, a limiting factor in the analysis of offside decisions through the evaluation of freeze images is that the impact of the players’ movements at the time of the offside decision cannot be considered. Especially for dynamic team sports as soccer it would be of great relevance to explore the attacking player’s movements in relation to the offside line, and also the defensive player’s movements (e.g., [8]). This should be considered in future research work.

If performance by soccer assistant referees is negatively influence by limited spatial attentional capacities, which is clarified by restricted visual angles, then officials might consider ways to compensate for these limits, thereby improving performance. Previous suggestions for optimized assistant referees’ decisions include using video replays (e.g., [14]), microchips (e.g., [15]), introducing new training programs [8], or adding perceptual information like additional lines to the field (e.g., [16]). The fact that there is a relationship between the attentional performance measurement in the laboratory and the misdeterminations of offside calls by assistant referees in the field depending on the wide of their visual angle, leads to the question if it is possible to train attentional spread, in order to optimize the decision behavior of assistant referees in offside situations in the long run. Future studies should take up these deliberations and could control possible changes in performance with the help of the attentional spread task used in Study 2.

Counterintuitively, our findings suggest that assistant referees might make more accurate calls if they were positioned further away from the action; from a more distant vantage point the relevant players in an offside call would be separated by a smaller visual angle, making it possible for the assistant referee to attend to the relevant players simultaneously. Unfortunately, doing so likely would be impractical given the possible consequences for other judgments assistant referees must make (e.g., out of bounds calls) and due to the variable layout of stadiums. This preliminary evidence that greater visual angles and smaller separations to the players in the depth are associated with increased errors in offside calls may explain the complaints of fans: Those seated directly behind the assistant referee, further from the players, might actually have it easier to make the right call.
